# Amine-Functionalized Porous Copolymeric Microspheres for Efficient Chromium(VI) Removal: Synthesis and Characterization

**DOI:** 10.3390/ma19102036

**Published:** 2026-05-13

**Authors:** Małgorzata Maciejewska, Grzegorz Wójcik

**Affiliations:** 1 Department of Polymer Chemistry, Institute of Chemical Sciences, Faculty of Chemistry, Maria Curie-Skłodowska University in Lublin, Gliniana 33, 20-614 Lublin, Poland; 2 Department of Inorganic Chemistry, Institute of Chemical Sciences, Faculty of Chemistry, Maria Curie-Skłodowska University in Lublin, M. Curie-Sklodowska Sq. 2, 20-031 Lublin, Poland; grzegorz.wojcik2@mail.umcs.pl

**Keywords:** porous polymer microspheres, epoxy-functional polymers, amine-modified adsorbents, chromium(VI) removal

## Abstract

Porous glycidyl methacrylate-based copolymers crosslinked with ethylene glycol dimethacrylate (EGDMA) and trimethylolpropane trimethacrylate (TMPTMA) were synthesized via suspension–emulsion polymerization and subsequently functionalized with triethylenetetramine. The effect of the monomer composition on the epoxy group content and porous structure was systematically investigated by varying the GMA-to-crosslinker molar ratio from 1:1 to 5:1. Increasing the GMA fraction enhanced the epoxy group content (2.8–5.0 mmol/g) but significantly reduced the specific surface area (333–23 m^2^/g), indicating a trade-off between functionality and porosity. ATR-FTIR and elemental analysis confirmed successful amine functionalization while preserving a considerable degree of porosity. The modified copolymers were evaluated for Cr(VI) removal, showing strong pH dependence, with maximum efficiency at pH 3 due to electrostatic interactions between protonated amine groups and HCrO_4_^−^ ions. Equilibrium studies revealed saturation-type behavior, with a maximum sorption capacity of 165.47 mg/g for TMPTMA-based copolymers. Despite the higher nitrogen content in EGDMA-based materials, TMPTMA-crosslinked copolymers exhibited a superior adsorption performance, demonstrating that pore accessibility, rather than functional group density alone, governs adsorption efficiency. These findings provide insight into the rational design of amine-functionalized porous polymer sorbents for efficient chromium(VI) removal.

## 1. Introduction

The increasing contamination of water resources by toxic heavy metals remains a serious environmental and public health concern. Among these pollutants, chromium in its hexavalent oxidation state (Cr(VI)) is particularly hazardous due to its high solubility, mobility, mutagenicity, and carcinogenicity [1,2,3,4,5]. Cr(VI) is widely discharged from electroplating, leather tanning, pigment production, textile dyeing, and metallurgical industries. In aqueous systems, chromium(VI) exists predominantly as oxyanions (HCrO_4_^−^, Cr_2_O_7_^2−^, and CrO_4_^2−^), and their relative distribution is strongly dependent on pH. Under acidic conditions, HCrO_4_^−^ is the dominant species, which facilitates Cr(VI) removal via electrostatic attraction to positively charged sorbent surfaces [6,7,8]. Therefore, the development of efficient adsorbents capable of selectively binding anionic chromium species in acidic media is of considerable importance. Among the various techniques used for heavy metal removal, adsorption is regarded as one of the most effective due to its operational simplicity, high efficiency, and economic viability. In this context, permanently porous polymer microspheres have attracted significant attention as advanced sorbent materials [9,10,11,12,13,14,15,16]. Polymer microspheres (also referred to as beads or microbeads) are spherical particles with diameters ranging from several tens of nanometers up to approximately 2000 µm, composed of one or more polymeric components [17]. Their well-defined geometry, mechanical stability, chemical resistance, and tunable surface chemistry make them particularly suitable for separation and purification processes [18,19,20].

Polymer microspheres can be synthesized via a variety of heterogeneous polymerization techniques, including suspension, precipitation, dispersion, emulsion, and seeded (co)polymerization. The selected method strongly influences the particle size, morphology, and internal structure [21,22]. Emulsion polymerization typically produces nanoparticles due to polymerization within surfactant-stabilized micelles, whereas suspension polymerization yields larger spherical microspheres formed within dispersed monomer droplets. Precipitation and dispersion polymerization allow for the preparation of relatively uniform microspheres without the need for large quantities of stabilizers. Importantly, when polymerization is conducted in the presence of appropriate porogenic solvents and multifunctional crosslinkers, phase separation during network formation leads to the development of permanent porosity. The resulting materials may exhibit hierarchical pore structures comprising micro-, meso-, and macropores, which are essential for achieving high surface areas and favorable mass-transfer properties in sorption applications [23,24]. However, achieving an optimal balance between high functional group density and well-developed porosity remains a significant challenge, as increasing the content of reactive monomer units often leads to reduced crosslinking density and deterioration of textural properties. These difficulties can be overcome by application of glycidyl methacrylate (GMA)-based copolymers [25,26,27,28]. The presence of reactive epoxy groups enables straightforward post-polymerization functionalization via ring-opening reactions with numerous nucleophilic reagents, including amines, thiols, lactams, sodium cyclopentadiene and alcohols, under relatively mild conditions [29,30,31,32,33,34]. In particular, the modification of epoxy-containing copolymers with polyamines represents an effective strategy for introducing a high density of nitrogen donor atoms into the porous matrix. In turn, copolymerization with multifunctional crosslinkers ensures the formation of rigid, insoluble networks [35,36]. Careful adjustment of the GMA-to-crosslinker molar ratio provides a powerful tool for tailoring the density of epoxy groups and, consequently, the extent of subsequent chemical modification [37]. Despite numerous reports on GMA-based sorbents, systematic investigations addressing the interplay between the monomer composition, epoxy content, porosity evolution, and final adsorption performance remain limited.

In this work, porous copolymers based on glycidyl methacrylate were synthesized via suspension—emulsion polymerization. Ethylene glycol dimethacrylate (EGDMA) and trimethylolpropane trimethacrylate (TMPTMA) were used as crosslinking agents, allowing for modulation of the network porosity due to their different functionalities and structural architectures. The molar ratio of GMA to crosslinker was systematically varied from 1:1 to 5:1 to investigate its influence on the epoxy group content and porous structure. The obtained microspheres were subsequently modified with triethylenetetramine (TETA) in order to introduce amine functionalities. The synthesized materials were thoroughly characterized with respect to their chemical compositions and textural parameters, and their performances in chromium(VI) removal from aqueous solutions were evaluated, with particular emphasis on adsorption under acidic conditions.

## 2. Materials and Methods

2,3-Epoxypropyl methacrylate (GMA), trimethylolpropane trimethacrylate (TMPTMA), and ethylene glycol dimethacrylate (EGDMA) (Sigma-Aldrich, Steinheim, Germany) were washed with 5% aqueous sodium hydroxide in order to remove inhibitors. Sodium bis(2-ethylhexyl) sulfosuccinate (AOT) and a,a’-azoisobutyronitrile (AIBN) purchased from Fluka AG (Buchs, Switzerland) were used without purification. Toluene, decan-1-ol, acetone, and methanol (reagent grade) were from POCh (Gliwice, Poland). Triethylenetetramine (TETA) was purchased from Tokyo Chemical Industry (Tokyo, Japan). The water for the experiment was prepared in the demineralization system Polwater DL-2, Kraków, Poland. The conductivity of the used water was 0.06 µS/cm. Standard solutions of chromium(VI) were prepared from the certified reference solution (1000 mg/L), produced by Romil (Cambridge, UK). The chromium(VI) solutions were prepared from the analytical-grade potassium salt K_2_Cr_2_O_7_—POCH Gliwice, Poland. The other reagents—NaOH and H_2_SO_4_, of analytical grade—were from Chempur, Piekary Śląskie, Poland.

Synthesis and Modification

**Figure 1:**
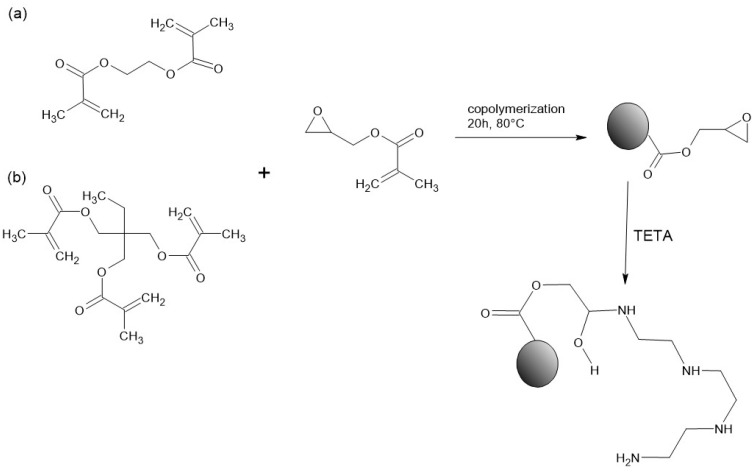
Scheme of copolymerization reaction of GMA with EGDMA (**a**) and TMPTMA (**b**) followed by modification with triethylenetetramine.

Porous copolymers in the form of regular microspheres were synthesized via suspension—emulsion polymerization in an aqueous medium. Initially, 2.2 g of AOT was dissolved in 195 mL of distilled water at 80 °C. Separately, a monomer mixture containing 15 g of monomers (GMA and selected crosslinker) together with 0.2 g of the radical initiator (AIBN) and 15 mL of pore-forming mixture (toluene + decan-1-ol) was prepared and subsequently introduced into the aqueous phase under continuous stirring. The molar ratio between the functional monomer and the crosslinking agent was adjusted over the range 1:1–5:1. The copolymerization reaction was conducted at 80 °C for 20 h. After synthesis, the particles were subjected to an extensive purification procedure, following protocols reported previously [20]. The concentration of epoxy functionalities within the copolymer network was quantified by back-titration. The epoxy groups present in the copolymer were modified using two-fold excess of TETA in relation to the epoxy group content. Figure 1 presents the general path of the synthesis and modification.

Measurement Methods

The porous characteristics of the copolymers were evaluated using an ASAP 2405 N surface area and porosity analyzer (Micromeritics Corp., Norcross, GA, USA). Nitrogen adsorption–desorption isotherms were recorded, and the specific surface area (S_BET_) was calculated from the linear region of the Brunauer–Emmett–Teller (BET) plots. The total pore volume was estimated from the amount of nitrogen adsorbed at a relative pressure of 0.985, while the pore size distribution was derived from the desorption branch of the isotherm using the Barrett–Joyner–Halenda (BJH) method.

The morphology of the copolymer microspheres was examined by scanning electron microscopy using a DualBeam™ Quanta 3D FEG microscope (FEI Corp., Hillsboro, OR, USA). Prior to imaging, the samples were coated with a thin gold layer to ensure adequate conductivity. All micrographs were acquired at an accelerating voltage of 5 kV.

The analysis of the size and distribution of the microspheres was made using a Mastersizer Analyser 2000 (Malvern, Instruments Ltd., Worcestershire, UK). The statistic of the distribution was calculated using the derived diameters in accordance with British standard BS2955:1993. According to this standard, D(0.1) is the size in microns of particles below which 10% of the sample lies, and D(0.9) is the size of particles below which 90% of the sample lies. D(0.5) refers to the Mass Median Diameter (MMD), that is, the size in microns at which 50% of the sample is smaller and 50% is larger. Span (width of the size distribution) was calculated as:

$$span=\frac{d(0.9)-d(0.1)}{d(0.5)}$$

Thermal analysis was performed to elucidate the influence of the copolymer composition and post-polymerization functionalization on the stability and degradation behavior of the porous microspheres. Thermogravimetric analyses were carried out on a Netzsch STA 449 F1 Jupiter thermal analyzer (Selb, Germany). Approximately 10 mg of each sample was placed in an alumina crucible and heated from ambient temperature to 700 °C at a heating rate of 10 K min^−1^ under a helium atmosphere. The resulting thermogravimetric curves were used to determine the characteristic decomposition temperatures corresponding to 20% and 50% mass losses, as well as the temperature of the maximum degradation rate (T_max_) for individual decomposition stages. The gaseous products evolved during thermal degradation were analyzed using a Fourier-transform infrared (FTIR) spectrometer coupled online to the STA system. FTIR spectra were collected at 10 °C intervals over a spectral range of 600–4000 cm^−1^, with a resolution of 4 cm^−1^.

Differential scanning calorimetry (DSC) measurements were performed using a DSC 204 instrument (Netzsch, Selb, Germany). Samples weighing 10 ± 0.2 mg were sealed in aluminum crucibles with pierced lids and heated from room temperature to 500 °C at a rate of 10 °C/min under an argon flow of 30 mL/min. An empty aluminum pan was used as a reference.

The DRS (Diffuse Reflectance Spectroscopy) spectra were measured using a Jasco V-660 spectrometer equipped with a diffuse reflectance attachment (PIV-756), produced by Jasco, Tokyo, Japan.

In the adsorption studies, 0.05 g of the modified copolymer was equilibrated with the 25 mL chromium(VI) solution in the concentration 100 mg/L in the shaking bath at 293 K. The samples were shaken within the 1440 min using the laboratory shaker Elpin+ type 358 (Lubawa, Poland) at amplitude 7 and speed 160 cpm. After the determined time, the samples were filtered, and the filtrates were analyzed. The UV-VIS method was used for determination of Cr(VI) ions, with 2% diphenylcarbazide solution. The absorbance was measured using a spectrophotometer, BK-UV 1900PC (Biobase, Shandong, China), at a wavelength of 543 nm (Figures S2 and S3).

The removal efficiency $R_{Cr(VI)}$ (%)) was calculated from Equation (1):

(1) $$R_{Cr(VI)}=\frac{C_{Cr(VI)}}{C_{i}}\cdot 100\%$$

where *C_i_* is the initial concentration of Cr(VI) in the solution (mg dm^−3^); $C_{Cr(VI)}$ is the concentration of Cr(VI) in the solution (mg dm^−3^) (calculated as the difference in the chromium(VI) ion concentrations in the solution before and after sorption).

The sorption capacity was calculated from Equation (2):

(2) $$q_{eCr(VI)}=\frac{{{(C}_{i}-C}_{eCr\left(VI\right)})}{m}V$$

where $C_{i}$ is the initial concentration of Cr(VI) in the solution (mg dm^−3^); $C_{eCr\left(VI\right)}$ is the equilibrium concentration of Cr(VI) in the solution (mg dm^−3^); ***m*** is the mass of the copolymer (g); ***V*** is the volume of the solution (dm^3^).

The adsorption isotherm experiment was carried out as follows: an amount of 0.05 g of resin with 25 mL of chromate solution in the concentration range 100–2000 mg/L was equilibrated at the laboratory temperature in the shaker bath for 1440 min.

## 3. Results

**Figure 2:**
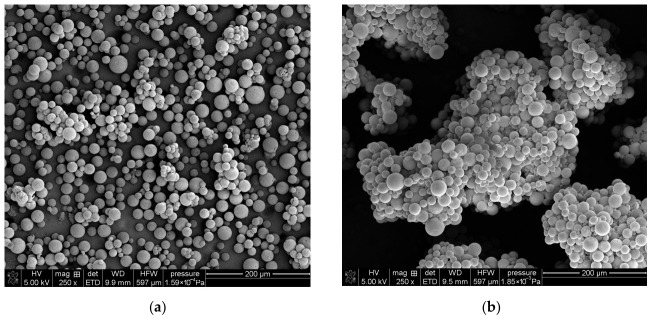
SEM images of synthesized microspheres: (**a**) poly(GMA-co-EGDMA)_1; (**b**) poly(GMA-co-TMPTMA)_1. Scale bar: 200 µm.

Porous poly(GMA-co-EGDMA) and poly(GMA-co-TMPTMA) microspheres were successfully synthesized by the suspension–emulsion polymerization technique. Unlike conventional suspension polymerization, where droplet stabilization is typically achieved using a high-molecular-weight protective colloid, the present system employed the anionic surfactant AOT as the stabilizing agent. Because the surfactant concentration in the aqueous phase exceeded its critical micelle concentration, polymerization was initiated and propagated within micellar aggregates while still yielding regular microspherical particles. The morphology of the synthesized copolymer microspheres was examined by SEM (Figure 2), while their particle size distribution was determined using laser diffraction analysis. The SEM images confirm that both poly(GMA-co-EGDMA)_1 and poly(GMA-co-TMPTMA)_1 were obtained in the form of regular, spherical particles with relatively smooth external surfaces, indicating the effective stabilization of the dispersed phase during polymerization.

The quantitative particle size analysis is presented in Table 1. The median particle sizes (D(0.5)) indicate that both copolymer systems produced microspheres of comparable size. However, differences in the width of the particle size distributions were observed. The calculated span values, equal to 1.11 for the EGDMA-based copolymer and 1.08 for the TMPTMA-based material, suggest a slightly narrower and more uniform particle size distribution in the latter case. The observed differences can be related to the functionality of the crosslinking agents. The trifunctional TMPTMA promotes faster network formation and earlier structural stabilization of the droplets, limiting their growth and leading to a more homogeneous particle population. In contrast, the difunctional EGDMA forms a more flexible network, allowing for greater droplet deformation and coalescence, which results in a broader size distribution.

To obtain microspheres with highly developed internal porous structures, a binary mixture of toluene and decan-1-ol was applied as the porogenic system. The use of mixed porogens is advantageous because each component plays a distinct role during polymerization and phase separation. Toluene acts as a relatively good solvent for the forming polymer chains at the early stages of polymerization, whereas decan-1-ol behaves as a poorer solvent, reducing polymer solubility as the reaction proceeds. This difference in solvent quality facilitates controlled phase separation during network formation, leading to the generation of a permanently porous internal structure. The porous architecture is formed when the growing copolymer chains reach a critical molecular mass and become insoluble in the reaction medium, resulting in the appearance of polymer-rich and polymer-lean domains. After removal of the porogenic solvents, the polymer-lean regions remain as pores, while the polymer-rich domains form the rigid crosslinked skeleton of the microspheres. In the present system, the binary porogen composition promoted a more favorable phase separation pathway than single-solvent systems, yielding materials with well-developed porosities and stable pore architectures. Importantly, the addition of decan-1-ol, whose solubility parameter (δ = 17.6 (MPa)^1/2^) is close to that of toluene (δ = 18.2 (MPa)^1/2^), also influenced the chemical composition of the resulting copolymer. Because the polarity of the porogenic medium became better adjusted to both the crosslinking monomer and glycidyl methacrylate, the distribution of GMA within the polymerizing droplets was improved, reducing compositional heterogeneity during copolymerization. As a consequence, GMA was incorporated more efficiently into the polymer matrix, which was confirmed by the higher concentration of epoxy groups in comparison with analogous materials synthesized previously using pure toluene as the sole porogen [29]. The molar ratio of the functional monomer was systematically varied from 1:1 to 5:1. This compositional variation had a pronounced influence on both the chemical functionality and textural characteristics of the resulting materials. In both copolymer series, increasing the GMA content led to a steady rise in the epoxy group concentration, confirming the more effective incorporation of the functional monomer into the copolymer framework (Table 2). Simultaneously, this compositional change was accompanied by a marked deterioration in the porous structure, reflected by the decreasing specific surface area and pore volume. In the poly(GMA-co-EGDMA) series, the epoxy group concentration increased from 2.87 to 5.02 mmol/g. However, this increase was accompanied by a 74% reduction in the specific surface area and a 63% decrease in the pore volume. In parallel, the pore diameter evolved from a bimodal distribution centered at 3.75 and 35 nm for the most highly crosslinked sample to approximately 40 nm for the sample containing the highest GMA fraction. These trends can be explained by phase separation dynamics during polymerization. A higher EGDMA content in the polymerization mixture promotes earlier phase separation and favors the formation of structures with smaller pores. In contrast, reduced the crosslinking density at higher GMA loadings delays phase separation, increases chain mobility, and promotes pore coarsening, leading to the partial collapse or merging of finer pores. These observations are consistent with results reported previously for analogous GMA-based porous copolymeric systems [29].

**Figure 3:**
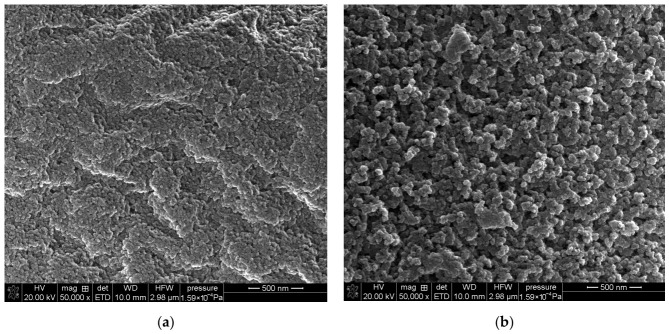
SEM images of porous surfaces of synthesized microspheres: (**a**) poly(GMA-co-EGDMA)_1; (**b**) poly(GMA-co-TMPTMA)_1.

An analogous trend was observed for the poly(GMA-co-TMPTMA) materials, although these copolymers displayed substantially more developed porosities than their EGDMA-based counterparts throughout the entire composition range. The specific surface area decreased from 333 to 86 m^2^/g with the increasing GMA content, while the epoxy group content rose from 1.45 to 4.23 mmol/g. The pore diameters simultaneously increased from 19 to 39 nm. Compared with the EGDMA series, the TMPTMA-crosslinked copolymers retained considerably higher surface areas and pore volumes at corresponding compositions, which indicates that the trifunctional crosslinker generates a more open and texturally stable porous network (Figure 3).

At the same time, the epoxy group contents in this series remained lower than those in the EGDMA-based analogues, suggesting that improved porosity is achieved at the expense of a lower concentration of reactive epoxy functionalities. Post-polymerization modification of the porous copolymers with triethylenetetramine resulted in the successful introduction of nitrogen-containing functional groups while preserving a measurable degree of porosity in all investigated materials. The textural parameters and elemental compositions of the modified microspheres are summarized in Table 3.

The obtained results indicate that the amination process affected not only the chemical composition of the copolymers but also their internal porous architectures, and that the magnitude of these changes strongly depended on both the initial monomer composition and the type of crosslinking agent used. For the poly(GMA-co-EGDMA)_TA series, a gradual increase in the nitrogen content from 6.3 to 8.9 wt.% was observed with the increasing GMA fraction in the precursor copolymers. This trend confirms that the extent of triethylenetetramine incorporation was directly related to the number of epoxy groups available for the nucleophilic ring-opening reaction. Since the initial epoxy group content increased systematically with increasing GMA content, a higher density of reactive sites enabled the more effective introduction of amine functionalities.

To quantitatively evaluate the efficiency of epoxy group conversion during amination, the theoretical nitrogen contents (assuming one TETA molecule per epoxy group and four nitrogen atoms per molecule) were compared with the experimentally determined values. The results are summarized in Table 4.

The results reveal that the functionalization efficiency is not constant but decreases with increasing epoxy group content. For both copolymer series, the efficiency converges to approximately 30–35% at higher degrees of functionalization, which can be attributed to steric hindrance and diffusion limitations within the porous network. At lower epoxy contents, higher efficiencies are observed, particularly for the TMPTMA-based materials, indicating better accessibility of reactive sites. Importantly, at comparable epoxy group levels, both copolymer systems exhibit similar functionalization efficiencies, confirming that the lower nitrogen content in TMPTMA-based materials results primarily from their lower initial epoxy concentration rather than reduced reactivity.

**Figure 4:**
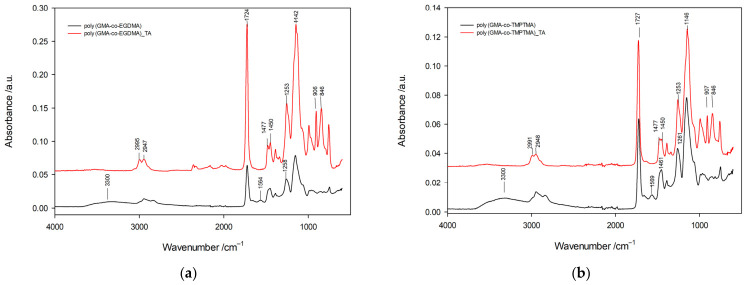
ATR-FTIR spectra of copolymers: (**a**) EGDMA-based copolymer and (**b**) TMPTMA-based copolymer before and after post-polymerization modification with triethylenetetramine.

The increase in the nitrogen content was accompanied by a pronounced deterioration in the textural parameters. The specific surface area decreased from 144 to 29 m^2^/g, while the pore volume declined from 0.581 to 0.170 cm^3^/g. These changes indicate that the growing degree of functionalization was associated with the partial filling or blocking of the porous structure by the attached triethylenetetramine chains. The pore diameter values provide additional insight into the structural consequences of the modification. For poly(GMA-co-EGDMA)_1_TA, a bimodal pore distribution was still observed, with maxima at approximately 4 and 38 nm, whereas in the subsequent samples, the average pore diameter shifted progressively toward larger values, reaching 41 nm for poly(GMA-co-EGDMA)_5_TA. This increase should not be interpreted as an improvement in porosity but rather as a consequence of the disappearance of smaller pores and the relative predominance of larger remaining voids. Such behavior is typical of systems in which narrow pores become inaccessible due to the chemical functionalization or partial collapse of less rigid regions of the polymer network. A similar tendency was observed for the poly(GMA-co-TMPTMA)_TA series, although the absolute textural parameters remained considerably more favorable than for the EGDMA-based analogues. In this group, the nitrogen content increased systematically from 5.4 to 7.8 wt.%, again confirming that higher GMA fractions promoted more extensive amination. Simultaneously, the specific surface area decreased from 303 to 79 m^2^/g. Although this reduction was significant, the final values remained substantially higher than those recorded for the corresponding EGDMA-crosslinked materials. Likewise, the pore volume remained relatively high throughout the series, varying from 0.604 to 0.421 cm^3^/g. The pore diameter increased from 21 to 40 nm, indicating a progressive reduction in fine porosity similar to that observed for the EGDMA-based copolymers. The superior preservation of porosity in the TMPTMA-crosslinked materials indicates that the trifunctional crosslinker generated a more rigid and structurally stable polymer network, capable of accommodating post-polymerization modification without the severe collapse of the internal porous framework. In contrast, the EGDMA-based materials, formed with a difunctional crosslinker, appear more susceptible to structural rearrangement during amination, particularly at high GMA contents where the overall crosslinking density is lower. This difference demonstrates that the choice of crosslinker determines not only the precursor porosity but also the resistance of the polymer architecture to subsequent chemical transformation. A direct comparison of both copolymer families reveals an important structure—property relationship. At corresponding monomer compositions, the EGDMA-based microspheres consistently exhibited higher nitrogen contents than the TMPTMA analogues, whereas the TMPTMA-based materials retained significantly larger specific surface areas and pore volumes. This means that the EGDMA series provided chemically richer sorbents with higher densities of amine functionalities, while the TMPTMA series offered superior accessibility of the porous structure. Such behavior reflects the characteristics of the precursor copolymers: higher epoxy group contents in the EGDMA series favored more extensive amination, whereas the more highly crosslinked TMPTMA networks better preserved permanent porosity. These observations are particularly important from the perspective of chromium(VI) adsorption. Although a higher nitrogen content increases the number of protonatable amine groups capable of binding chromate ions, efficient sorption also requires that these functional groups remain accessible within the porous network. Excessive reduction of the internal surface area may limit diffusion of Cr(VI) oxyanions into the polymer matrix and reduce the effective utilization of chemically introduced adsorption sites. Therefore, the final adsorption performance cannot be predicted solely on the basis of the nitrogen content. Instead, the most efficient sorbent is expected to be the material in which the density of amine groups is balanced by sufficient pore accessibility. The quantitative trends further support this interpretation. In the EGDMA series, the nitrogen content increased by approximately 41%, whereas the specific surface area decreased by nearly 80%. In the TMPTMA series, the nitrogen content increased by approximately 44%, while the specific surface area decreased by about 74%. Although both systems exhibited comparable relative gains in nitrogen functionality, the TMPTMA-based microspheres retained markedly higher absolute porosity after modification. This indicates that TMPTMA provides a more robust framework for introducing amine groups without excessive loss of structural accessibility. However, textural data alone do not provide direct evidence of the chemical transformation occurring during modification. Therefore, ATR-FTIR spectroscopy was used to examine the structural changes associated with the reaction of epoxy groups with triethylenetetramine. The ATR-FTIR spectra of the copolymers before and after post-polymerization modification with triethylenetetramine are presented in Figure 4.

The spectra of the unmodified materials exhibit the characteristic absorption bands of crosslinked methacrylate copolymers containing glycidyl methacrylate units. In both copolymer series, a strong absorption band observed at ca. 1724 cm^−1^ is assigned to the stretching vibrations of ester carbonyl groups (C=O), originating from both GMA and the crosslinking monomers. The bands located in the range 2995–2947 cm^−1^ correspond to stretching vibrations of aliphatic C-H bonds, while the signals observed at ca. 1450–1477 cm^−1^ and 1388 cm^−1^ are attributed to deformation vibrations of -CH_2_ and -CH_3_ groups. The bands at ca. 1253 and 1142 cm^−1^ are associated with C-O and C-O-C vibrations of ester groups present in the polymer network. Particular attention should be paid to the absorption bands at ca. 906 and 846 cm^−1^, which are characteristic of epoxy ring vibrations originating from glycidyl methacrylate units. These signals constitute the most important spectroscopic evidence of the presence of reactive epoxy functionalities in the precursor copolymers and confirm that the polymerization process preserved the oxirane groups required for subsequent chemical modification. After treatment with triethylenetetramine, distinct spectral changes were observed in both copolymer systems, indicating successful post-polymerization functionalization. The intensity of the epoxy-related bands at ca. 906 and 846 cm^−1^ decreased markedly, which confirms the consumption of epoxy groups during nucleophilic ring-opening reactions with amine groups. At the same time, new absorption features appeared in the spectral regions characteristic of nitrogen-containing functionalities. In particular, a broad band centered around 3300 cm^−1^ can be attributed to overlapping N–H and O–H stretching vibrations. This broadening is consistent with the formation of amino-alcohol structures generated during epoxy ring opening, in which each reaction step introduces both nitrogen-containing groups and hydroxyl functionalities into the polymer matrix. Additional evidence of successful amination is provided by the appearance of a band at ca. 1568 cm^−1^, assigned to deformation vibrations of N–H groups. Furthermore, an increase in intensity in the region around 1258 cm^−1^ can be related to C–N vibrations and partially to C–O vibrations of newly formed hydroxyl-containing structures. Importantly, the strong ester carbonyl band at ca. 1725 cm^−1^ remained unchanged after modification, indicating that the main polymer backbone was preserved and that the reaction occurred selectively at epoxy sites without affecting the ester framework of the copolymer. Analogous spectral changes were observed for the poly(GMA-co-TMPTMA) copolymers after modification with triethylenetetramine, confirming that the reaction mechanism was independent of the crosslinking monomer used. In both copolymer families, the reduction in the epoxy band intensity accompanied by the appearance of amine-related absorption bands demonstrates that triethylenetetramine was successfully incorporated into the porous polymer network. The spectroscopic observations are fully consistent with the elemental analysis results, which show a significant increase in nitrogen content after modification. Taken together, these results confirm that the post-polymerization reaction proceeded via the nucleophilic attack of amine groups on the epoxy rings, leading to the formation of covalently bonded amino functionalities rather than simple physical adsorption of the modifying agent. From the viewpoint of adsorption performance, the introduction of amine groups is particularly important because under acidic conditions, they become protonated, generating positively charged active centers capable of strong electrostatic interaction with chromium(VI) oxyanions. The thermal stability of the synthesized porous copolymers was investigated by thermogravimetric analysis under a helium atmosphere, and the characteristic decomposition temperatures are summarized in Table 5.

**Figure 5:**
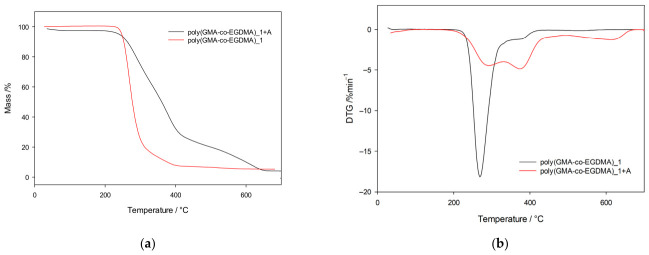
TG and DTG curves: (**a**) EGDMA-based copolymer and (**b**) TMPTMA-based copolymer before and after post-polymerization modification with triethylenetetramine.

Both poly(GMA-co-EGDMA) and poly(GMA-co-TMPTMA) copolymers exhibited two main degradation stages, reflected by the presence of two maxima on the DTG curves (T_1max_ and T_2max_), which is characteristic of highly crosslinked methacrylate-based porous polymers (Figure 5).

For the poly(GMA-co-EGDMA) series, increasing the GMA content resulted in a gradual decrease in the thermal stability. The temperature corresponding to 5% mass loss (T5%) decreased from 250 to 236 °C, while T50% decreased from 287 to 266 °C with the increasing GMA fraction. Simultaneously, the first maximum decomposition temperature (T_1max_) shifted from 273 to 253 °C. This behavior indicates that increasing the concentration of glycidyl methacrylate units reduces the thermal resistance of the polymer network. The observed trend can be attributed to the lower crosslinking density and increased flexibility of the copolymer structure at higher GMA contents. In addition, epoxy-containing fragments are thermally less stable than densely crosslinked methacrylate networks, which further contributes to earlier thermal degradation. A similar tendency was observed for the poly(GMA-co-TMPTMA) copolymers; however, these materials exhibited significantly higher thermal stabilities over the entire composition range. In particular, the T_20%_ and T50% values were markedly higher than those of the corresponding EGDMA-based materials. For poly(GMA-co-TMPTMA)_1, the T_50%_ value reached 353 °C, compared with only 287 °C for the analogous EGDMA-crosslinked copolymer. Moreover, the second maximum decomposition temperatures (T_2max_) for the TMPTMA series were observed in the range 438–461 °C, approximately 90 °C higher than those of the EGDMA-based copolymers. The superior thermal stability of the TMPTMA-crosslinked materials can be attributed to the trifunctional nature of the crosslinking monomer, which generates a more rigid and highly interconnected three-dimensional network. The enhanced crosslinking density restricts chain mobility and increases resistance toward thermal decomposition. Importantly, the higher thermal stability of the TMPTMA-based copolymers correlates well with their superior preservation of the porous structure observed in nitrogen adsorption measurements. This indicates that the highly crosslinked TMPTMA framework provides not only enhanced thermal resistance but also improved structural stability of the porous architecture. The thermal stability of the TETA-functionalized copolymers was investigated by thermogravimetric analysis under a helium atmosphere, and the characteristic decomposition temperatures are summarized in Table 6. In comparison with the precursor copolymers, the modified materials exhibited significantly altered thermal behavior, indicating that post-polymerization amination strongly affected the structure and stability of the polymer network. For the poly(GMA-co-EGDMA)_TA series, the temperatures corresponding to 20% and 50% mass losses increased substantially after modification.

In particular, the T_50%_ values increased from 266–287 °C for the precursor copolymers to 360–369 °C after amination. Similarly, the T_20%_ values increased markedly, especially for copolymers containing lower GMA fractions. These results indicate that the reaction of epoxy groups with triethylenetetramine enhanced the thermal resistance of the polymer matrix. The improved thermal stability can be attributed to the conversion of epoxy groups into amino-alcohol structures during nucleophilic ring-opening reactions. The introduction of hydroxyl and amine functionalities increases intermolecular interactions within the polymer network, particularly hydrogen bonding, which restricts segmental mobility and delays thermal decomposition. In addition, the consumption of thermally less stable epoxy groups contributes to the shift of decomposition processes toward higher temperatures. Despite the overall improvement in the thermal resistance after modification, a gradual decrease in characteristic decomposition temperatures with increasing GMA content was still observed within each copolymer series. This behavior is associated with the reduced crosslinking density at higher GMA fractions, which results in the increased flexibility of the polymer framework and lower resistance toward thermal degradation.

The poly(GMA-co-TMPTMA)_TA copolymers exhibited considerably higher thermal stabilities than the corresponding EGDMA-based materials throughout the entire composition range. The T_50%_ values reached 388–393 °C for the TMPTMA-based copolymers, significantly exceeding those of the EGDMA analogues. Moreover, the second maximum decomposition temperatures (T_2max_) were observed in the range 403–419 °C, confirming the superior thermal resistance of the TMPTMA-crosslinked networks. The enhanced stability of the TMPTMA-based copolymers is related to the trifunctional nature of the crosslinking monomer, which generates a more rigid and highly interconnected three-dimensional structure. Importantly, these materials also retained higher porosity after amination, indicating that the structurally robust TMPTMA network effectively preserves both the thermal stability and porous architecture during post-polymerization modification. Overall, the thermal analysis demonstrates that both the crosslinking density and the degree of post-polymerization modification strongly influence the stability of porous copolymer networks. The results further confirm that TMPTMA provides a more thermally and structurally stable framework for the preparation of amine-functionalized porous sorbents.

**Figure 6:**
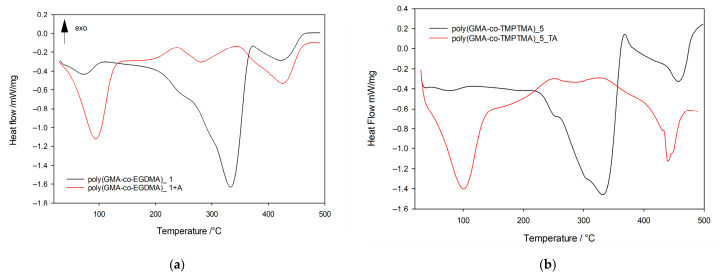
DSC curves: (**a**) EGDMA-based copolymer and (**b**) TMPTMA-based copolymer before and after post-polymerization modification with triethylenetetramine.

The influence of amine functionalization on the thermal behavior of the copolymers was further investigated by differential scanning calorimetry (DSC). The incorporation of nitrogen-containing groups significantly affects the thermal stability, degradation pathways, and intermolecular interactions within the polymer matrix. Figure 6 presents the DSC curves of the unmodified copolymer and its derivative modified with triethylenetetramine.

The DSC profile of the precursor copolymer exhibits exclusively endothermic thermal events. The initial endothermic peak observed below 100 °C is associated with the removal of physically adsorbed water and corresponds to an enthalpy change of approximately −32 J·g^−1^. The principal thermal transition occurs above 200 °C and consists of two overlapping endothermic peaks. These transitions closely correspond to the temperatures of the maximum mass loss rate determined by thermogravimetric analysis, indicating that they are related to the main stages of the thermal degradation of the copolymer network. A further endothermic effect observed above 400 °C is attributed to the decomposition of residual carbonaceous degradation products formed during earlier decomposition stages. In contrast, the DSC curve of the modified poly(GMA-co-EGDMA)_1_TA copolymer reveals substantially different thermal behavior, demonstrating the pronounced effect of amine incorporation on the physicochemical properties of the material. Unlike the unmodified copolymer, the modified material exhibits both endothermic and exothermic thermal events. The initial endothermic peak appears in a similar temperature range; however, its enthalpy value increases significantly to approximately 233 J·g^−1^. This enhanced thermal effect indicates a markedly higher affinity toward water, resulting from the presence of hydrophilic amine and hydroxyl groups introduced during epoxy ring-opening reactions. These functionalities promote hydrogen bond interactions with water molecules, increasing the moisture sorption capacity of the modified copolymer. The first exothermic transition, with a maximum near 270 °C, followed by a gradual evolution into an endothermic signal, is attributed to thermally induced reactions between residual epoxy groups and amine functionalities, leading to additional crosslinking within the polymer matrix. The subsequent endothermic effect corresponds to the degradation of the modified polymer network and the formation of reactive intermediate species. These intermediates likely participate in secondary thermally induced reactions, reflected by a broad exothermic peak centered around 370 °C. Similarly to the precursor material, a final endothermic transition above 400 °C is associated with the ultimate decomposition of the highly crosslinked polymer backbone and the degradation of residual charred structures. Analogical thermal behavior was observed for poly(GMA-co-TMPTMA) copolymers (Figure 6b). Overall, the DSC analysis confirms that post-polymerization amination significantly modifies the thermal response of the copolymers by increasing intermolecular interactions, altering degradation pathways and enhancing the hydrophilic character of the polymer network.

**Figure 7:**
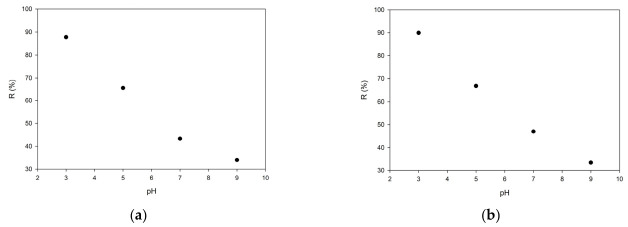
pH effects on removal efficiency of Cr(VI) ions on TETA-functionalized copolymers: (**a**) EGDMA-based copolymer and (**b**) TMPTMA-based copolymer.

In the next step of the investigation, the modified copolymers were used in the sorption of Cr(VI). In strongly acidic media, where HCrO_4_^−^ is the dominant chromium species, protonated amino groups can effectively bind chromate ions through electrostatic attraction and ion exchange interactions. At the same time, ATR-FTIR results indicate that the modification did not lead to the complete disappearance of epoxy-related bands, suggesting that a fraction of epoxy groups remained unreacted. This observation is consistent with the porous and crosslinked nature of the materials, in which part of the epoxy groups may remain sterically inaccessible within the internal polymer network. Such partial conversion is typical for highly crosslinked porous copolymers and indicates that the modification efficiency is governed not only by the epoxy group concentration but also by the accessibility of reactive sites within the pore system. These results are particularly important when considered together with the textural data. The chemical introduction of amine groups increases the number of active adsorption sites, whereas the simultaneous partial reduction in porosity may influence the accessibility of these sites to chromium(VI) ions. The final sorption performance of the modified materials depends on achieving an optimal balance between nitrogen functionality and preservation of the internal porous structure. Consequently, for the sorption studies, poly(GMA-co-EGDMA)_4_TA and poly(GMA-co-TMPTMA)_4_TA were chosen. The effects of the solution pH on the removal efficiency of Cr(VI) ions for both EGDMA- and TMPTMA-based copolymers are presented in Figure 7.

In both cases, a pronounced dependence of the adsorption efficiency on the pH was observed. The removal efficiency increased systematically with decreasing pH, reaching maximum values of 89.96% and 87.78% at pH 3 for the EGDMA- and TMPTMA-based copolymers, respectively. This behavior can be explained by considering both the speciation of chromium(VI) in aqueous solutions and the protonation state of amine groups introduced during post-polymerization modification. At alkaline pH, chromium(VI) is present predominantly as CrO_4_^2−^ ions, while amine groups remain largely unprotonated, resulting in weak electrostatic interactions and relatively low removal efficiencies (~33–34%). As the pH decreases, chromium(VI) speciation shifts toward HCrO_4_^−^ ions, which are more readily adsorbed due to their lower charge. Simultaneously, amine groups become progressively protonated, forming positively charged –NH_3_^+^ sites that strongly attract anionic chromium species. As a result, a significant increase in adsorption efficiency is observed, particularly in the acidic region.

**Figure 8:**
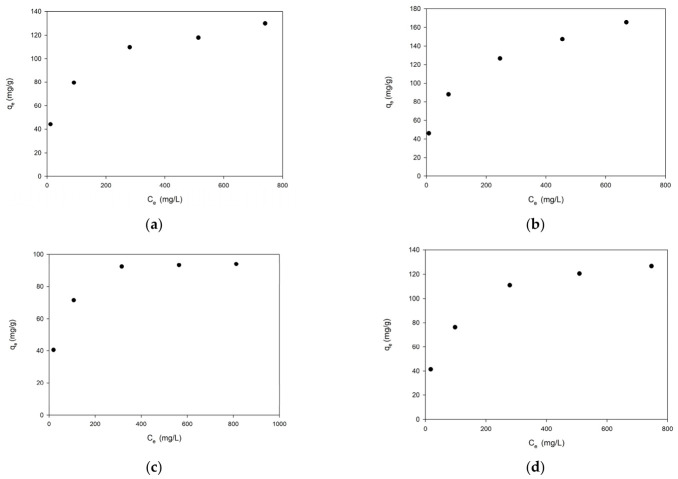
Isotherms of Cr(VI) ion sorption on TETA-functionalized copolymers: (**a**) EGDMA-based copolymer and (**b**) TMPTMA-based copolymer in 0.1 M H_2_SO_4_, and (**c**) EGDMA-based copolymer and (**d**) TMPTMA-based copolymer at pH 3.

Despite the differences in the textural properties and nitrogen contents between the two copolymer systems, their adsorption efficiencies are remarkably similar over the entire pH range. The EGDMA-based copolymers, characterized by higher nitrogen contents, provide a greater density of amine functional groups, whereas the TMPTMA-based materials retain a more developed porous structure and higher surface area after modification. The comparable removal efficiencies observed for both systems indicate that these two factors compensate each other. While a higher amine content favors the increased availability of adsorption sites, improved porosity enhances the accessibility of these sites to Cr(VI) ions. The highest removal efficiency observed at pH 3 confirms that electrostatic interactions between protonated amine groups and HCrO_4_^−^ ions constitute the dominant adsorption mechanism. The equilibrium adsorption behavior of Cr(VI) ions on the modified copolymers was investigated in 0.1 M H_2_SO_4_ and at pH 3, and the obtained isotherms are presented in Figure 8.

The adsorption capacity (q_e_) increased with the increasing equilibrium concentration (C_e_) for both EGDMA- and TMPTMA-based copolymers, followed by a gradual approach to saturation at higher concentrations. For the TMPTMA-based copolymers, the adsorption capacity increased from 45.99 mg/g at a low equilibrium concentration to a maximum value of 165.47 mg/g. In contrast, the EGDMA-based materials exhibited a lower maximum adsorption capacity of approximately 129.66 mg/g. In both cases, the shape of the isotherms suggests a typical saturation behavior, indicating the progressive occupation of available adsorption sites. The observed adsorption profiles are consistent with Langmuir-type behavior, suggesting that Cr(VI) adsorption occurs predominantly via monolayer coverage on a finite number of energetically equivalent active sites. These sites are associated with protonated amine groups introduced during post-polymerization modification with triethylenetetramine. At low equilibrium concentrations, a rapid increase in the adsorption capacity is observed due to the high availability of active sites and strong electrostatic attraction between protonated amine groups and chromium(VI) oxyanions. As the concentration increases, the adsorption process gradually approaches saturation, indicating that the number of available binding sites becomes limiting. A comparison of the two copolymer systems reveals that, despite the higher nitrogen content of the EGDMA-based materials, their adsorption capacity is significantly lower than that of the TMPTMA-based copolymers. This result indicates that the density of functional groups alone does not determine the adsorption performance. Instead, the accessibility of these groups, governed by the porous structure of the material, plays a decisive role. The TMPTMA-based copolymers, which retained higher specific surface areas and pore volumes after modification, provide more accessible adsorption sites, enabling more efficient interaction with Cr(VI) ions. In contrast, the EGDMA-based materials, although richer in amine functionalities, exhibit reduced porosity and limited accessibility of active sites due to partial pore blocking and structural densification during modification. As a result, a significant fraction of amine groups may remain inaccessible to chromium species, leading to a lower overall adsorption capacity. These findings demonstrate that the adsorption performance of amine-functionalized porous copolymers is governed by a balance between functional group density and structural accessibility. The superior performance of the TMPTMA-based materials indicates that preservation of the porous architecture is more critical than maximizing the number of functional groups alone. The high adsorption capacity observed under strongly acidic conditions further confirms that electrostatic interactions between protonated amine groups and HCrO_4_^−^ ions constitute the dominant adsorption mechanism. The results are consistent with the pH-dependent behavior and ATR-FTIR analysis, which confirmed the successful incorporation of amine functionalities into the polymer structure. The calculated values of the sorption parameters of Cr(VI) ions are presented in Table 7.

As follows from the results in Table 7, the Langmuir model (Equation (S5)) describes Cr(VI) sorption in a better way than the Freundlich one (Equation (S6)). The values of the R^2^ for the Langmuir model are about 0.996 and are higher than those of the Freundlich model.

The measured total capacity values were compared with literature data and are presented in Table 8.

As can be seen from the table, the sorption capacities obtained for chromium(VI) ions indicate the high efficiency of the sorbents. TMPTMA is characterized by a particularly high sorption capacity, reaching 134.72 mg/g.

**Figure 9:**
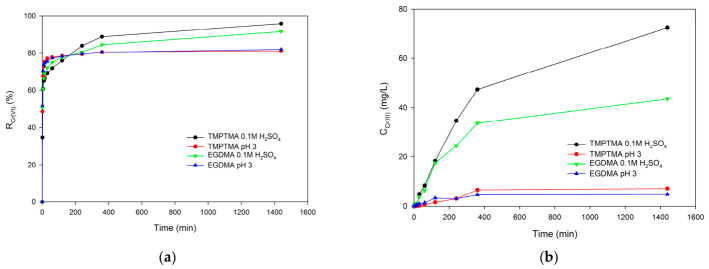
Effect of contact time on removal efficiency of Cr(VI) (**a**); concentration of Cr(III) during chromium(VI) ion sorption (**b**). Initial concentration of Cr(VI) = 100 mg/L.

Kinetic studies were conducted to estimate the rate of Cr(VI) ion adsorption (Figure 9, Equations (S3) and (S4)). Since amino groups can reduce chromium(VI) ions to chromium(III), the concentration of Cr(III) ions in the solution was also measured.

As can be seen from Figure 9, both Cr(VI) and Cr(III) forms were determined during the kinetic experiment. For up to 200 min, the %R values are higher for the solution with a pH of 3, while after 200 min, these values are higher in the 0.1 M sulfuric acid solution. This is due to the reduction of Cr(VI) ions to Cr(III). As can be seen from Figure 9b, the concentration of chromium(III) ions in the solution is low at pH 3, while in the 0.1 M sulfuric acid solution, it increases to 72.4 mg/L for TMPTMA and to 43.5 mg/L for EGDMA after 1440 min. The values of the kinetic parameter calculated using the pseudo-first- and pseudo-second-order models of sorption are presented in Table 8.

**Figure 10:**
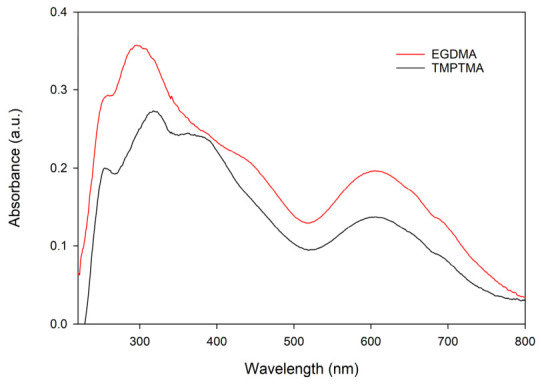
DRS spectra of Cr(VI) and Cr(III) ions after sorption on TETA-functionalized copolymers: EGDMA-based copolymer and TMPTMA-based copolymer in 0.1 M H_2_SO_4_.

The data presented in Table 9 indicate that the pseudo-second-order model of sorption describes the kinetics of sorption of Cr(VI) ions in a better way than the pseudo-first-order one. Since chromium(III) ions are present in the solution, the solid-phase sorbents EGDMA 0.1 M H_2_SO_4_ and TMPTMA 0.1 M H_2_SO_4_ were analyzed using the DRS method. The obtained spectra are presented in Figure 10. The band between 600 and 640 nm is associated with the presence of chromium(III) in the solid phase of the sorbent.

As can be seen in Figure 10, the band intensity of Cr(III) is lower for TMPTMA than that for EGDAMA. This is due to the higher concentration of chromium(III) ions in the solution for TMPTMA than for EGDAMA, as shown in the sorption kinetics studies (Figure 9).

**Figure 11:**
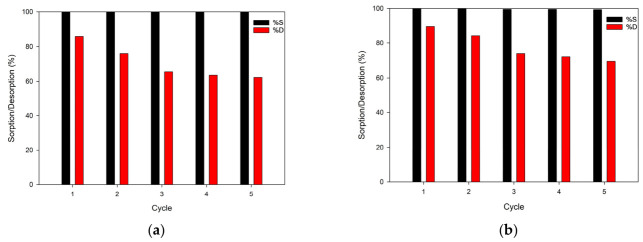
Sorption and desorption cycle efficiencies of Cr(VI) ions on TETA-functionalized copolymers: (**a**) EGDMA-based copolymer; (**b**) TMPTMA-based copolymer. Initial concentration of Cr(VI) = 10 mg/L.

Desorption of ions after the sorption process is very important and should be conducted with high efficiency. For the desorption process of Cr(VI) ions, the 0.1 M NaCl + 0.1 M NaOH solution was used. The results of the desorption are presented in Figure 11.

The results of the sorption and desorption tests showed that the desorption efficiency decreased with each cycle of work. Since the reduction of Cr(VI) ions to Cr(III) was detected, this may have resulted in a decrease in the concentration of chromium(VI) ions after desorption. In addition, high efficiency in the sorption of chromium(VI) ions was observed over the next five operating cycles.

Based on the results obtained, the following mechanism of chromium(VI) can be proposed:

EGDMA-NH + H^+^ + HCrO_4_^−^ = EGDMA-NHH^+^HCrO_4_^−^

TMPTMA-NH + H^+^ + HCrO_4_^−^ = TMPTMA-NHH^+^HCrO_4_^−^

2EGDMA-NH + H^+^ + CrO_4_^2−^ = (EGDMA-NHH^+^)_2_CrO_4_^2−^

2TMPTMA-NH + H^+^ + CrO_4_^2−^ = (TMPTMA-NHH^+^)_2_CrO_4_^2−^

EGDMA-NH_2_ + H^+^ + HCrO_4_^−^ = EGDMA-NH_2_ H^+^HCrO_4_^−^

TMPTMA-NH_2_ + H^+^ + HCrO_4_^−^ = TMPTMA-NH_2_ H^+^HCrO_4_^−^

2EGDMA-NH_2_ + H^+^ + CrO_4_^2−^ = (EGDMA-NH_2_ H^+^)_2_CrO_4_^2−^

2TMPTMA-NH_2_ + H^+^ + CrO_4_^2−^ = (TMPTMA-NH_2_ H^+^)_2_CrO_4_^2−^

As can be seen from the above reactions, the mechanism involves the protonation of amino groups and electrostatic interactions with chromate anions.

The Gibb’s free energy (∆G°) values for Cr(VI) sorption on TETA-functionalized copolymers were calculated from the equations presented in the Supplementary Materials (Equations (S1) and (S2), Figures S4 and S5). The calculated values of the thermodynamic parameters are presented in Table 10.

The negative values of the ∆G° show the spontaneity of the Cr(VI) adsorption process. The positive values for standard enthalpy show an endothermic process. The positive entropy change (∆S°) reflects an increase in disorder at the solid—liquid interface during adsorption. A similar thermodynamic effect was observed for chromium(VI) ion sorption on amide- and amidoxime-functionalized polymers with amine groups [38]. Also, graphene oxide/polyamidoamine dendrimer (GO/PAMAM) composites were used to remove Cr(VI) with positive values of the ∆S° and ∆H° [42].

## 4. Conclusions

Porous glycidyl methacrylate-based copolymers crosslinked with EGDMA and TMPTMA were successfully synthesized via a suspension–emulsion polymerization approach and subsequently functionalized with triethylenetetramine. The applied synthetic strategy enabled the preparation of regular microspheres with tunable epoxy group contents and porous structures, depending on the monomer composition and type of crosslinker used. Systematic variation in the GMA-to-crosslinker ratio revealed a clear trade-off between the functional group density and textural properties. Increasing the GMA content led to a significant increase in the epoxy group concentration, which enhanced the efficiency of the post-polymerization amination but simultaneously resulted in a pronounced decrease in the specific surface area and pore volume. In contrast, TMPTMA-crosslinked copolymers exhibited more developed and structurally stable porous networks compared with EGDMA-based materials, demonstrating that the nature of the crosslinker plays a crucial role in preserving porosity. ATR-FTIR analysis confirmed the successful modification of the copolymers through the nucleophilic ring opening of epoxy groups by triethylenetetramine, as evidenced by the reduction in characteristic epoxy bands and the appearance of signals corresponding to amine functionalities. Elemental analysis further verified the effective incorporation of nitrogen-containing groups into the polymer matrix.

The modified copolymers exhibited high efficiency in chromium(VI) removal from aqueous solutions, particularly under strongly acidic conditions. The adsorption process was strongly pH-dependent, with maximum removal efficiency observed at pH 3, which can be attributed to the combined effect of chromium speciation and protonation of amine groups. Under these conditions, electrostatic interactions between protonated amine sites and HCrO_4_^−^ ions dominate the adsorption mechanism. Equilibrium studies demonstrated that the adsorption behavior follows a saturation-type profile characteristic of monolayer adsorption. The maximum sorption capacity reached 165.47 mg/g for TMPTMA-based copolymers, which was significantly higher than that of the EGDMA-based materials (~129.66 mg/g), despite their lower nitrogen content. The obtained values of the sorption capacity are higher than most values presented in the literature. Additionally, the studied sorbents are characterized by fast kinetics of sorption. After 5 min of contact time, the values of the %R exceed more than 60%. The negative values of the ∆G° show the spontaneity of the Cr(VI) adsorption process. The suspected process of Cr(VI) reduction to Cr (III) was confirmed both in the solid phase as well as in solution. Importantly, the conducted investigation proved the possibility of the regeneration of the studied materials by using 0.1 M NaOH + 0.1 M NaCl solution.

Overall, the results demonstrate that the optimal design of amine-functionalized polymer sorbents requires a balance between high functional group density and preservation of accessible porosity. The superior performance of TMPTMA-based materials highlights the importance of crosslinker selection in controlling both structural stability and adsorption efficiency. The developed materials show strong potential as effective sorbents for chromium(VI) removal from acidic aqueous solutions.

## Figures and Tables

**Table 1:** Statistical data on particle size distribution.

Copolymer	D(0.1) (µm)	D(0.5) (µm)	D(0.9) (µm)	Span
poly(GMA-co-EGDMA)_1	18	27	48	1.11
poly(GMA-co-TMPTMA)_1	13	24	39	1.08

**Table 2:** Textural properties and epoxy group contents of microspheres synthesized at different molar ratios of monomers.

Copolymer	Specific Surface Area (*S*_BET_) (m^2^/g)	Pore Volume (*V*) (cm^3^/g)	Pore Diameter (*D_BJH_*) (nm)	Epoxy Group Number (mmol/g)
poly(GMA-co-EGDMA)_1 poly(GMA-co-EGDMA)_2 poly(GMA-co-EGDMA)_3 poly(GMA-co-EGDMA)_4 poly(GMA-co-EGDMA)_5	151 78 62 44 39	0.601 0.453 0.414 0.274 0.220	3.75/35 * 30 31 32 40	2.87 3.56 4.53 4.86 5.02
poly(GMA-co-TMPTMA)_1 poly(GMA-co-TMPTMA)_2 poly(GMA-co-TMPTMA)_3 poly(GMA-co-TMPTMA)_4 poly(GMA-co-TMPTMA)_5	333 182 99 90 86	0.694 0.665 0.405 0.537 0.461	19 25 31 38 39	1.45 2.61 3.15 3.84 4.23

* Bimodal distribution.

**Table 3:** Textural properties of microspheres after post-polymerization modification with triethylenetetramine.

Copolymer	Specific Surface Area (*S*_BET_) (m^2^/g)	Pore Volume (*V*) (cm^3^/g)	Pore Diameter (*D_BJH_*) (nm)	Nitrogen Content (wt. (%))
poly(GMA-co-EGDMA)_1_TA poly(GMA-co-EGDMA)_2_TA poly(GMA-co-EGDMA)_3_TA poly(GMA-co-EGDMA)_4_TA poly(GMA-co-EGDMA)_5_TA	144 73 59 38 29	0.581 0.402 0.381 0.234 0.170	4/38 * 33 34 37 41	6.3 7.4 7.7 8.1 8.9
poly(GMA-co-TMPTMA)_1_TA poly(GMA-co-TMPTMA)_2_TA poly(GMA-co-TMPTMA)_3_TA poly(GMA-co-TMPTMA)_4_TA poly(GMA-co-TMPTMA)_5_TA	303 162 91 80 79	0.604 0.585 0.387 0.462 0.421	21 29 34 38 40	5.4 6.4 6.7 7.3 7.8

* bimodal distribution.

**Table 4:** Comparison of theoretical and experimental nitrogen contents and functionalization efficiencies of TETA-modified copolymers.

Copolymer	Theoretical N (mmol/g)	Experimental N (wt.%)	Experimental N (mmol/g)	Efficiency (%)
poly(GMA-co-EGDMA)_1_TA poly(GMA-co-EGDMA)_2_TA poly(GMA-co-EGDMA)_3_TA poly(GMA-co-EGDMA)_4_TA poly(GMA-co-EGDMA)_5_TA	11.48 14.24 18.12 19.44 20.08	6.3 7.4 7.7 8.1 8.9	4.50 5.28 5.49 5.78 6.35	39.2 37.1 30.3 29.7 31.6
poly(GMA-co-TMPTMA)_1_TA poly(GMA-co-TMPTMA)_2_TA poly(GMA-co-TMPTMA)_3_TA poly(GMA-co-TMPTMA)_4_TA poly(GMA-co-TMPTMA)_5_TA	5.80 10.44 12.60 15.36 16.92	5.4 6.4 6.7 7.3 7.8	3.85 4.57 4.78 5.21 5.57	66.4 43.8 37.9 33.9 32.9

**Table 5:** Thermal stabilities of parent copolymers determined in helium.

Copolymer	T_5%_ (°C)	T_20%_ (°C)	T_50%_ (°C)	T_1max_ (°C)	T_2max_ (°C)
poly(GMA-co-EGDMA)_1 poly(GMA-co-EGDMA)_2 poly(GMA-co-EGDMA)_3 poly(GMA-co-EGDMA)_4 poly(GMA-co-EGDMA)_5	250 246 245 245 236	264 258 260 260 248	287 277 278 280 266	273 266 270 270 253	370 368 369 369 367
poly(GMA-co-TMPTMA)_1 poly(GMA-co-TMPTMA)_2 poly(GMA-co-TMPTMA)_3 poly(GMA-co-TMPTMA)_4 poly(GMA-co-TMPTMA)_5	251 242 241 237 234	306 283 283 271 269	353 331 332 328 323	327 325 321 297 296	461 456 457 455 438

**Table 6:** Thermal stabilities of modified copolymers determined in helium.

Copolymer	T_5%_ (°C)	T_20%_ (°C)	T_50%_ (°C)	T_1max_ (°C)	T_2max_ (°C)
poly(GMA-co-EGDMA)_1_TA poly(GMA-co-EGDMA)_2_TA poly(GMA-co-EGDMA)_3_TA poly(GMA-co-EGDMA)_4_TA poly(GMA-co-EGDMA)_5_TA	254 249 247 247 239	301 298 290 260 248	369 368 368 366 360	306 294 293 301 292	379 378 369 370 369
poly(GMA-co-TMPTMA)_1_TA poly(GMA-co-TMPTMA)_2_TA poly(GMA-co-TMPTMA)_3_TA poly(GMA-co-TMPTMA)_4_TA poly(GMA-co-TMPTMA)_5_TA	263 254 253 252 250	316 307 304 303 292	393 392 389 388 366	336 301 297 302 288	419 418 411 419 403

**Table 7:** Freudlich and Langmuir isotherm model constants for chromium(VI) ion sorption.

**Freudlich Isotherm Parameters**			
	K_f_ (mg/g)	n	R^2^
EGDMA pH 3	22.21	4.37	0.9431
EGDMA 0.1 M H_2_SO_4_	25.14	4.04	0.9883
TMPTMA pH 3	18.51	3.33	0.9833
TMPTMA 0.1 M H_2_SO_4_	24.29	3.27	0.9890
**Langmuir Isotherm Parameters**			
pH	Qo (mg/g)	b (L/mg)	R^2^
EGDMA pH 3	97.96	0.03164	0.9996
EGDMA 0.1 M H_2_SO_4_	128.52	0.02267	0.9962
TMPTMA pH 3	134.72	0.01662	0.9966
TMPTMA 0.1 M H_2_SO_4_	174.99	0.02038	0.9962

**Table 8:** Comparison of sorption capacities of TMPTMA-based and EGDMA-based copolymers with other materials.

Material	Sorption Capacity [mg/g]	The Literature
AN-amidoxime polymer	28.83	[38]
AN-DMAPA polymer	130	[38]
AN-DMAPA polymer	120.2	[38]
PS-DMAPA resin	71.15	[39]
Amberlite IRA-400	69.9	[40]
Dowex PSR-2	121.95	[8]
Diaion CR20	169.49	[41]
EGDMA	97.96	Present Work
TMPTMA	134.72	Present Work

**Table 9:** Rate constants for chromium(VI) ion removal; initial concentration of Cr(VI) = 100 mg/L.

**First-Order Rate Constant**			
pH	k_1_ (1/min)	q_1_ (mg/g)	R^2^
EGDMA pH 3	0.00419	4.43	0.9354
EGDMA 0.1 M H_2_SO_4_	0.00431	14.26	0.9877
TMPTMA pH 3	0.00384	3.38	0.8347
TMPTMA 0.1 M H_2_SO_4_	0.00559	19.46	0.9912
**Second-Order Rate Constant**			
pH	k_2_ (g·(mg/min))	q_2_ (mg/g)	R^2^
EGDMA pH 3	0.00767	41.02	0.9999
EGDMA 0.1 M H_2_SO_4_	0.00177	45.88	0.9992
TMPTMA pH 3	0.01129	40.61	0.9999
TMPTMA 0.1 M H_2_SO_4_	0.00136	48.14	0.9991

**Table 10:** Thermodynamic parameters for Cr(VI) ion sorption on TETA-functionalized copolymers.

Parameter	∆G° (kJ/mol)			∆H° (kJ/mol)	∆S° (J/mol·K)
	298 K	308 K	328 K		
EGDMA-based	−11.58	−12.39	−13.40	6.20	60.60
TMPTMA-based	−12.51	−13.27	−14.26	4.55	58.12

## Data Availability

The original contributions presented in this study are included in the article. Further inquiries can be directed to the corresponding author.

## Supplementary Materials

The following supporting information can be downloaded at: https://www.mdpi.com/article/10.3390/ma19102036/s1, Figure S1: FTIR spectrum of poly(GMA-co-TMPTMA)_1_TA copolymer, Figure S2: Calibration curve of chromium(VI) ions at 543 nm, y = 0.724x + 0.0048, R2 = 0.9998. Figure S3: Calibration spectra of chromium(VI) ions. Figure S4: The isotherm of Cr(VI) ions sorption on TETA-functionalized copolymers: (a) EGDMA-based (b) TMPTMA-based at pH 3. Figure S5: Plots obtained on the basis of Vant´Hoof equations to determine the thermodynamic parameters of adsorption of Cr(VI) ions sorption on TETA-functionalized copolymers: (a) EGDMA-based (b) TMPTMA-based at pH 3.
