# Supplementary material for: Amine-Functionalized Porous Copolymeric Microspheres for Efficient Chromium(VI) Removal: Synthesis and Characterization
Source: Materials (Basel). 2026 May 13;19(10):2036. doi: 10.3390/ma19102036 (PMC13208387; doi:10.3390/ma19102036)
Supplement: Supplementary file 1 [file materials-19-02036-s001.zip › materials-4259426-supplementary.pdf]

Article - Supplementary Materials

Amine-Functionalized Porous Copolymeric Microspheres for Efficient Chromium(VI) Removal: Synthesis and Characterization

Małgorzata Maciejewska[1,*], Grzegorz Wójcik[2]

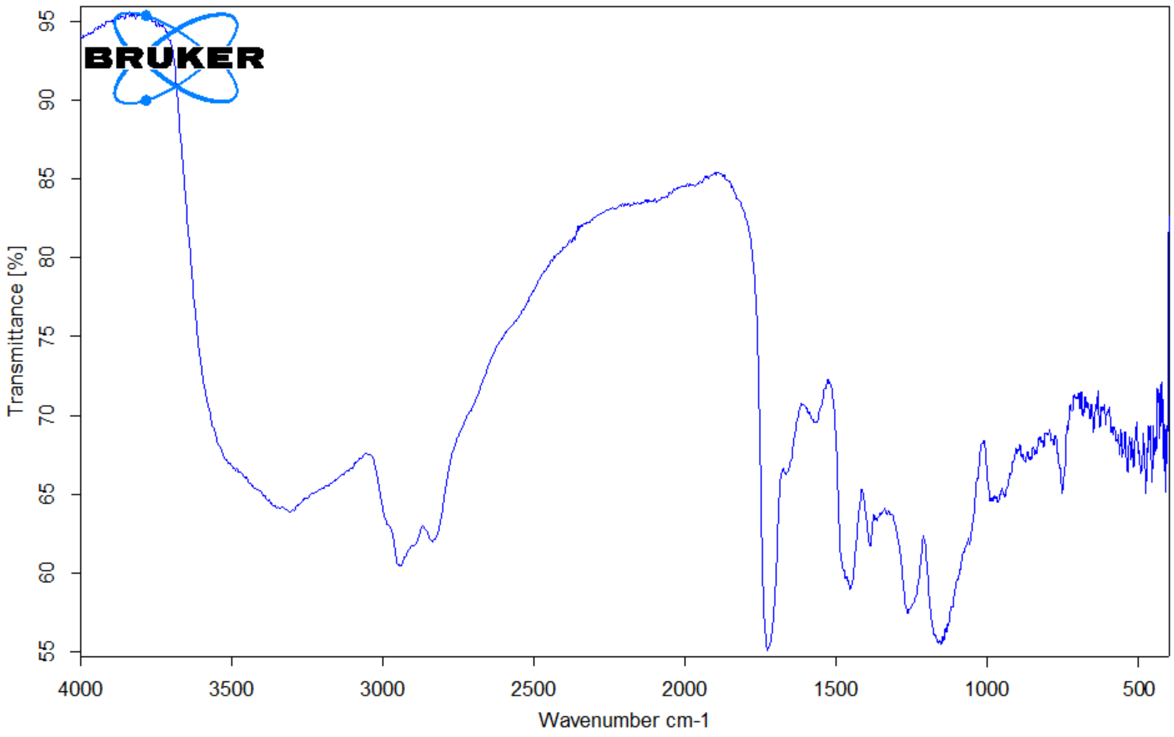

Figure S1. FTIR spectrum of poly(GMA-*co*-TMPTMA)_1_TA copolymer

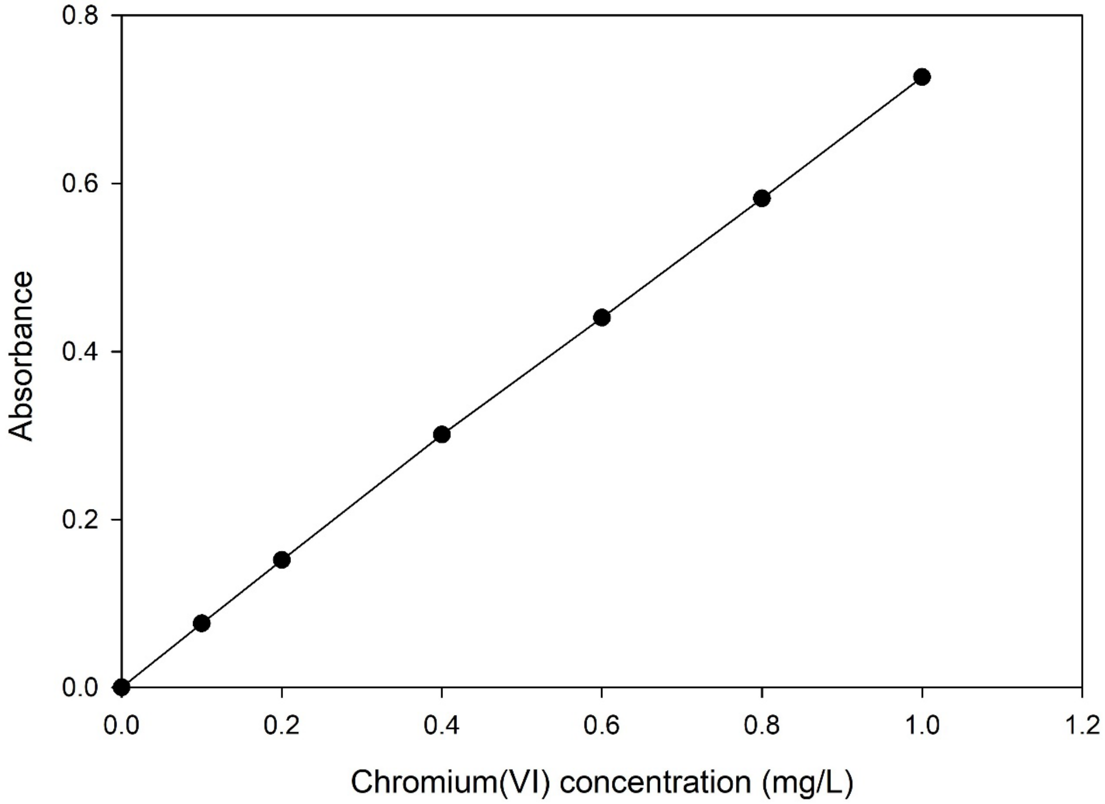

Figure S2. Calibration curve of chromium(VI) ions at 543nm, y=0.724x+0.0048, R^2=0.9998.

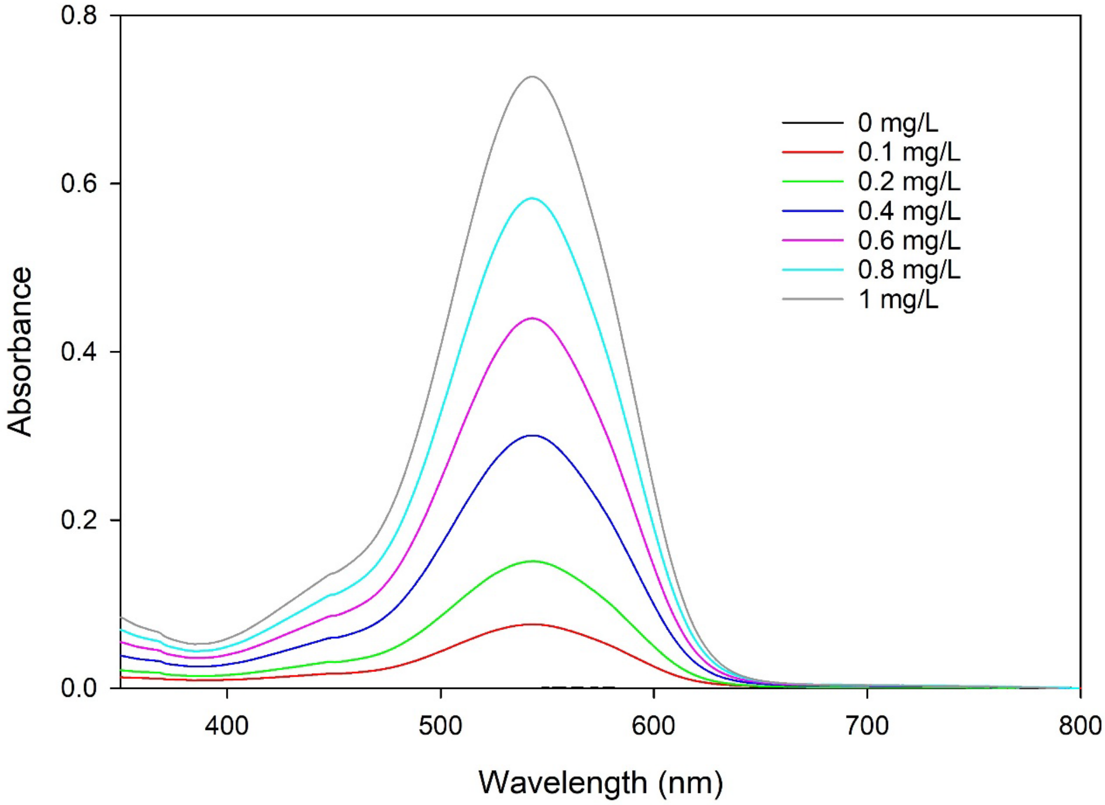

Figure S3. Calibration spectra of chromium(VI) ions.

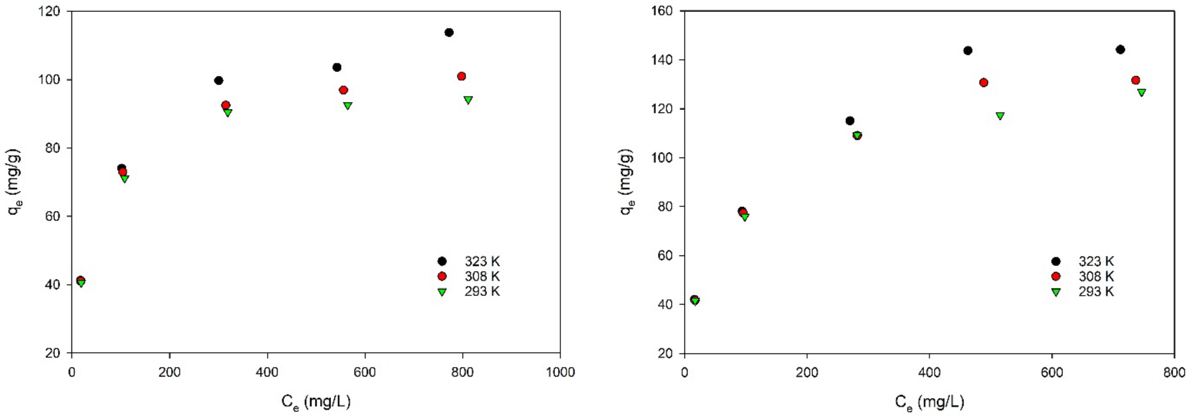

(a) (b)

Figure S4. The isotherm of Cr(VI) ions sorption on TETA-functionalized copolymers: (a) EGDMA-based (b) TMPTMA-based at pH 3 .

Thermodynamic parameters (enthalpy change ΔH°, entropy change ΔS° and Gibbs energy change ΔG°) were calculated based on the following equations :

$$\Delta G^{\circ} = -RT \ln K_c \quad \text{(S1)}$$

$$ln(K_c) = -\frac{\Delta H^{\circ}}{RT} + \frac{\Delta S^{\circ}}{R} \qquad (S2)$$

where R – the universal gas constant (8.314 J/mol・K), T – the temperature (K) and K_c – the equilibrium constant,

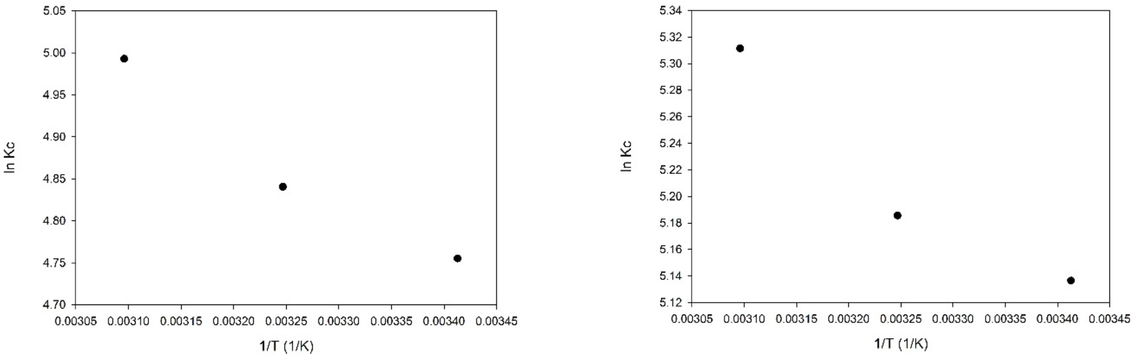

(**a**) (**b**)

Figure S5. Plots obtained on the basis of Vant´Hoof equations to determine the thermodynamic parameters of adsorption of Cr(VI) ions sorption on TETA-functionalized copolymers: (**a**) EGDMA-based (**b**) TMPTMA-based at pH 3

To determine the kinetics of adsorption the pseudo-first order chemical sorption (Eq. (S3)) and the pseudo-second order chemical sorption (Eq. (S4)) were used:

$$\frac{dq_t}{d_t} = k_1(q_1 - q_t) \qquad (S3)$$

where, k1 is the equilibrium rate constant of the pseudo-first order (min^{-1}), q1 is the amount of Cr(VI) sorbed at equilibrium ($mg{\cdot}g^{-1}$), qt is the amount of Cr(VI) sorbed at time t ($mg{\cdot}g^{-1}$).

$$\frac{dq_t}{d_t} = k_2(q_2 - q_t)^2 \qquad (S4)$$

where, k_2 is the equilibrium rate constant of the pseudo-second order ($g{\cdot}(mg{\cdot}min)^{-1}$), q2 is the amount of Cr(VI) sorbed at equilibrium ($mg{\cdot}g^{-1}$), q_t is the amount of Cr(VI) sorbed at time t ($mg{\cdot}g^{-1}$).

The Langmuir isotherm was calculated according to equation (S5):

$$\frac{C_e}{q_e} = \frac{1}{b \cdot Q_0} + \frac{C_e}{Q_0} \qquad \text{(S5)}$$

where, q_e is the amount of adsorbate in the adsorbent ($mg \cdot g^{-1}$); Ce is the equilibrium concentration ($mg \cdot L^{-1}$); b is the Langmuir isotherm constant ($L \cdot mg^{-1}$); Q_0 is the maximum monolayer coverage capacity ($mg \cdot g^{-1}$).

The Freundlich isotherm was calculated according to equation (S6)

$$\log q_e = \log K_F + \frac{1}{n} \log C_e \qquad \text{(S6)}$$

where, q_e is the amount of adsorbate in the adsorbent ($mg \cdot g^{-1}$); K_F is the characteristic constant related to the adsorption capacity ($mg \cdot g^{-1}$); n is the adsorption intensity; C_e is the equilibrium concentration ($mg \cdot L^{-1}$).
